# Climate Justice Strategies Implemented by Public Health Nurses and Their Community Partners

**DOI:** 10.1111/jan.16598

**Published:** 2024-11-11

**Authors:** Jessica LeClair, Alex Dudek, Susan Zahner

**Affiliations:** ^1^ School of Nursing University of Wisconsin—Madison Madison Wisconsin USA; ^2^ School of Medicine and Public Health University of Wisconsin—Madison Madison Wisconsin USA

**Keywords:** climate justice, community, health equity, health promotion, health services research, planetary health, population health, public health nursing, qualitative approaches, social justice

## Abstract

**Aim:**

To describe nurses' and community‐based organization representatives' collaborative strategies for advancing climate justice with communities.

**Design:**

This study used a descriptive, qualitative research design.

**Methods:**

Data were gathered from August 2022 to February 2023 with nurses (*n* = 8) and their community partners (*n* = 5) in the United States. Community partners were representatives of community‐based organizations. Photovoice provided greater context for the thematic analysis of collaborative strategies discussed in semi‐structured interviews.

**Results:**

Nurse participants worked in academic or non‐profit settings. Nurse‐community partnerships addressed corporate pollution and promoted Indigenous sovereignty and multispecies justice. Themes included investigating disease and health events, identifying at‐risk populations and connecting them with resources, providing health teaching and counseling, organizing communities and coalitions, and advocating for policy development and enforcement. Self‐care supported resilience and well‐being in the long struggle for climate justice.

**Conclusion:**

Findings from this study indicated that nurses and their community partners strategize to transition communities away from systems of extraction towards local and regenerative systems that support resilience. Nurses and their community partners recognized the importance of applying an expansive understanding of climate justice, including intersections of pollution and multispecies justice, to promoting planetary health.

**Implications for the profession:**

Findings from this study support nurse‐community collaboration in policy work to advance planetary health. This study also supports nurses' collective action with their community partners to address the effects of white supremacy and colonization. Future research is needed to evaluate the outcomes of nurse‐community partnerships for planetary health.

**Impact:**

Nurses have called for action on climate justice; however, evidence of effective nursing strategies that advance climate justice is sparse. This study is the first to describe the collaborative strategies nurses implement with community partners to support the transition from injustice to justice in communities most burdened by climate change and industrial pollution.

## 1 Introduction

The average global temperature is on track to surpass 1.5°C above preindustrial levels, bringing widespread devastation (Masson‐Delmotte et al. 2021). Climate change is the most severe public health threat of the 21st century and causes inequitable health impacts across different populations (World Health Organization [WHO] 2021). Climate justice connects the causes and effects of climate change to structural injustices in society (International Climate Justice Network 2002). Low‐income and racialised communities are most at risk for inequitable climate health impacts, which are climate injustices (Radavoi 2015). International and national professional nursing organisations recognise the urgency of addressing climate health inequities and have published calls to action (American Nurses Association [ANA] 2022, 2023; Nurses, Climate Change, and Health 2018; ‘Nursing Collaborative on Climate Change and Health’ 2017). Despite these calls for action, evidence of effective nursing strategies that advance climate justice is sparse (LeClair, Watts, and Zahner 2021).

Public health nursing in the United States is defined as promoting and protecting population health at local, state and national levels using knowledge from nursing, social and public health sciences (American Public Health Association [APHA] 2013). Public health nurses are a part of the most trusted health profession and practice in communities that experience health inequities (Brenan 2023). These nurses could be practical facilitators of climate justice information and interventions for affected communities. Public health nurses and community‐based organisation (CBO) representatives are expected to partner on various public health issues, including promoting environmental justice and planetary health (ANA 2022, 2023; APHA 2013; Kulbok et al. 2012). Evidence about climate justice strategies nurses implement in partnership with CBO representatives is needed. This study aimed to describe nurses' and CBO representatives' collaborative strategies for advancing climate justice with communities.

## 2 Background

Nurses who want to advance climate justice must understand the inequitable population health impacts of climate change in racialised and low‐income communities and the history of the climate justice movement in response to these impacts. Understanding communities' experiences on the frontlines of climate change impacts and the fencelines of toxic industries can inform nursing strategies to advance climate justice for public and planetary health (Evans‐Agnew, LeClair, and Sheppard 2024). A brief description of the existing literature about climate injustices, the climate justice movement, and climate justice in nursing follows.

### 2.1 Climate Injustices Worsen Public and Planetary Health

Climate change operates globally and manifests in local health issues, posing significant challenges for nurses (Anaker et al. 2015). Local impacts on the United States include the direct effects of heat waves, flooding and changes in the frequency and intensity of other extreme weather events (Jay et al. 2023). Climate change indirectly affects population health through degraded air quality, such as climate‐caused wildfire smoke. These factors cause an increased incidence of asthma exacerbation and increased risk of morbidity and mortality for those with cardiovascular disease (Hayden et al. 2023). Population health threats from climate change that nurses face also include adverse birth outcomes, injuries and premature deaths related to extreme weather events, increased number and kinds of infectious diseases and threats to mental health (Hayden et al. 2023; Usher et al. 2023). People who are pregnant, very young, very old and those who have disabilities or chronic health conditions experience more adverse impacts on health due to climate change, and those with decreased access to health care services experience even worse outcomes (Hayden et al. 2023).

Communities affected by environmental racism experience disproportionate exposure to environmental hazards (e.g., pollution and climate change) and associated health issues (Malin and Ryder 2018). Dr. Bullard (1994) defined environmental racism as ‘…any policy, practice, or directive that differentially affects or disadvantages (whether intended or unintended) individuals, groups, or communities based on race or color’ (p. 1037). Low‐income communities that experience environmental racism are often referred to as ‘frontline’ and ‘fenceline’ communities. Frontline communities are the first to experience climate crises, such as displacement from sea‐level rise (Garibay and Arevalo 2017). Fenceline communities live close to industrial or toxic environments and experience harm from the associated pollution (Radavoi 2015). Only recently have scholars begun considering the intersections of frontline and fenceline communities, such as the effects of industrial accidents precipitated by extreme weather due to climate change (Johnston and Cushing 2020).

Many nonhuman species also experience climate injustice in the form of habitat loss and extinction (Masson‐Delmotte et al. 2021). The transdisciplinary science and movement of planetary health focuses on understanding and addressing the impacts of human disruptions of Earth's ecological systems on human health and all life (Faerron Guzmán et al. 2021). Climate injustices intersect with these disruptions, causing problems such as decreasing healthy freshwater and forest ecosystems and catastrophic biodiversity loss. Multispecies justice is a term that expands the idea and practice of justice to encompass and respond to the destruction of multispecies ways of life and rejects the notion of human exceptionalism (Celermajer et al. 2020). The climate crisis is a threat to all life, inflicting unjust burdens on marginalised and displaced peoples, other species and their interconnected ecosystems.

### 2.2 The Climate Justice Movement

In response to the previously discussed injustices, climate justice emerged from the environmental justice movement over two decades ago to redefine climate change as a civil rights issue (International Climate Justice Network 2002). Climate justice remains a top priority of the environmental justice movement (Baptista et al. 2022). Formal Climate Justice Principles were published in 2002 for the *Earth Summit* in Bali (International Climate Justice Network 2002). Policy recommendations were published that same year at the *Second National People of Color Environmental Leadership Summit* (Miller and Sisco 2002). Further recommendations were put forth at a national Mobilization for Climate Justice when a climate justice community delegation attended the 2009 United Nations Climate Change Conference in Copenhagen (‘Mobilization for Climate Justice: Open Letter to the Grassroots’, 2009). During this time, the Just Transition Alliance was formed to seek a just transition from dangerous workplaces and environments to healthy and sustainable ones for workers and communities (The Just Transition Alliance Definition of a Just Transition and Just Transition Principles 1997). The climate justice movement continues to evolve and strengthen through the work of groups such as the Climate Justice Alliance, which was formed in 2013 to unite frontline communities in working towards resilient, regenerative and equitable economies (Climate Justice Alliance 2024).

### 2.3 Climate Justice in Nursing

Members of the Alliance of Nurses for Healthy Environments Global Climate Justice in Nursing Steering Committee utilised the previously described principles from the climate justice and planetary health movements to guide the development of the Global Nurse Agenda for Climate Justice to help inform climate justice strategies in nursing research, education, advocacy and practice (Evans‐Agnew, LeClair, and Sheppard 2024). The Global Nurse Agenda for Climate Justice outlined 36 principles to help guide nursing actions to advance climate justice and expanded international interest in nursing partnerships for climate justice and planetary health.

### 2.4 Public Health Nursing

Climate injustice significantly threatens frontline and fenceline communities' ecosystems and population health. Public health nurses promote and protect population health at local, state and national levels (ANA 2022; APHA 2013). A national study was completed with 176 public health nursing administrators, where they identified increasing climate and health concerns, including disruptions of health care services during weather events and population displacements due to floods and wildfires because of climate change (Polivka, Chaudry, and Mac Crawford 2012). Public health nurses address social injustices in the environments where people live, learn, work and play and are therefore called to advance climate justice in partnership with the communities they serve (ANA 2022; LeClair, Evans‐Agnew, and Cook 2022; Lilienfeld et al. 2018; Nicholas and Breakey 2017; Travers et al. 2019). A CBO is driven by residents in all aspects (National Community‐Based Organization Network 2004), and it is expected for nurses and CBOs to partner on public health issues (ANA 2022; APHA 2013; Kulbok et al. 2012). Yet, there remains a lack of published information on nurse and CBO collaborative strategies to advance climate justice in frontline and fenceline communities (LeClair, Watts, and Zahner 2021; Lilienfeld et al. 2018; Nicholas and Breakey 2017; Polivka and Chaudry 2018). Nursing research must identify effective strategies to advance climate justice through community partnerships.

### 2.5 The Study

This article presents the results of a research project conducted to describe collaborative strategies nurses and CBO representatives use for advancing climate justice with frontline and fenceline communities. The findings of this study provide evidence for effective collaborative strategies to address climate justice in communities.

## 3 Methods

### 3.1 Design

This study used a qualitative, descriptive research design to obtain and thematically analyse different and complementary data through photovoice and semistructured interviews.

#### 3.1.1 Theoretical Frameworks

The public health intervention wheel, the social ecological model and the critical environmental justice nursing for planetary health framework guided this study. The public health intervention wheel is a population‐based model encompassing three levels of practice (community, systems and individual/family) and 17 population‐based interventions (Schaffer and Strohschein 2019). The model displays these interventions in an interactive wheel, and each comprises various actions that public health nurses take in collaboration with individuals, families and communities to improve or protect health status.

Like the public health intervention wheel, the levels of the social ecological model are interactive and reinforcing, so they have a cumulative effect on population health (Golden and Wendel 2020). The social ecological model's core tenets are designed around five levels of influence on health, including intrapersonal factors, interpersonal processes, institutional factors, community factors and public policy (Golden and Wendel 2020). The model shifts away from an individual biomedical paradigm and instead recognises the social and structural determinants of population health equity.

The study also incorporates a critical lens by using the critical environmental justice nursing for planetary health framework (EJ nursing framework), which applies critical theories to the way people conceptualise the root causes of climate injustices as patterns of domination that degrade all life (LeClair, Luebke, and Oakley 2021). The EJ nursing framework was utilised to inform the development of interview guides to understand how nurses and CBOs collaboratively strategised to promote climate justice.

#### 3.1.2 Study Setting and Recruitment

The study population included nurses and their CBO partners. Criterion sampling was utilised to select participants (Polit and Beck 2017). To assist with criterion sampling, a link to a screening survey was emailed to nurses who were members of the Council of Public Health Nursing Organizations and state‐level nursing (*n* = 49 states and 2 territories), public health (*n* = 54) and climate and health organisations (*n* = 18) via their organisation email lists, newsletters or on their websites. Snowball recruiting was also used when some organisation members shared the opportunity with nurses who were not members or sent the first author contact information for the nurses they believed met the inclusion criteria for the study.

Fifty‐two nurses were self‐screened as eligible to participate through the recruitment survey, and there were eight additional snowball referrals for potential nurse participants. Of these, 31 nurses did not respond to multiple outreach attempts. Twenty‐one nurses declined to enrol without providing a reason, cited lack of time given current work or were ineligible due to lack of a suitable CBO partner or not being an RN during their experience working to support climate justice. The final sample was 13 participants, which included eight nurses (13% of eligible participants) and five CBO representative partners. Three of the eight nurses participated without a CBO partner. The nurses then shared this opportunity to participate with their community partners. The primary incentive was $150 for participating in the photovoice and semistructured interviews. Study participants were in Massachusetts, New Jersey, Wisconsin, Michigan, Ohio and Washington.

#### 3.1.3 Inclusion Criteria

Nurses were included in the study if they were (1) registered nurses, (2) identified through the recruitment survey as engaging in the practice of promoting and protecting the health of populations and (3) self‐identified as having experience partnering with a CBO to advance climate justice in a frontline/fenceline community. CBO representatives were included in the study if they were (1) identified by the nurses as community partners and (2) self‐identified as having experienced partnering with the nurse to advance climate justice in a frontline/fenceline community. If a CBO representative could not participate in the study, the nurse could enrol alone for partial data collection. Finally, nurses and CBO representatives were included if they volunteered to participate in the study.

#### 3.1.4 Data Collection

Nurses and CBO participants confirmed their interest in study participation during a video conference with the first author, during which signed consent was obtained and individuals were enrolled. Data were gathered from August 2022 to February 2023 through photovoice and semistructured, in‐depth, one‐on‐one interviews with nurses and their community partners. Photovoice interviews refer to the process by which photographs taken by participants were discussed using an adaptation of the photovoice method, which allows for photo contextualisation by having participants prioritise photos for discussion and answer specific questions for each chosen photo (Wang and Burris 1997). Photovoice was used to understand participants' climate justice experiences by asking them to take photographs of what climate justice looks like and means to them and then present and collaboratively analyse the photos and narratives in an interview session. Each nurse and CBO partner pair presented and analysed their photographs in interview sessions with the first author. The full results of the photovoice interviews, including descriptions of participants' climate justice perspectives and experiences, are reported elsewhere (LeClair 2023). While participants were not explicitly asked about strategies during the photovoice sessions, many discussed their implemented or suggested climate justice strategies, so all photovoice transcripts were included in the analysis. Semistructured interviews were also conducted with each participant to specifically discuss their strategies to advance climate justice. Initially, open‐ended, generative questions were used, and interview processes reflected a critical approach through an inquiry into systems of injustice that led to climate injustices (LeClair, Luebke, and Oakley 2021) (see Supporting Information for the full interview guides).

All interviews were held virtually using a HIPAA compliant video‐conferencing platform. The interviews were digitally recorded, downloaded with a password‐protected computer, transcribed verbatim via Zoom transcription by hired graduate students and checked for accuracy by the research team. All transcripts were returned to participants for comments or corrections.

#### 3.1.5 Data Analysis

Thematic analysis was implemented with eight photovoice transcripts (i.e., one for each partnership) and 13 semistructured interview transcripts (i.e., one for each participant). The research team followed Braun and Clarke's (2006) six phases of thematic analysis, including (1) becoming familiar with the data, (2) generating initial codes, (3) searching for themes, (4) reviewing themes, (5) defining and naming themes and (6) producing the report. Two research team members independently listened to the recordings, read the transcripts and wrote memos to facilitate analytic insights. Initial thematic codes were generated deductively from the previously described public health intervention wheel and social ecological model. This deductive analysis was implemented because these frameworks have been utilised by public health nurses and nurse educators worldwide since 2001 to implement strategies that address health inequities and promote population health (Schaffer, Strohschein, and Glavin 2022). The public health intervention wheel provides a common language for public health nurses to communicate about their practice with each other and external partners (Schaffer and Strohschein 2019). We also remained open to coding the data inductively when information emerged for potential themes not represented in the selected frameworks. Rigour was maintained through intercoder agreement within the research team and member checking with study participants. Codes from both the photovoice and semistructured interviews were combined into one codebook. Research reflexivity was also implemented using peer debriefing throughout the coding process and reflexive writing to keep an account of the research process and memo personal reflections throughout the study (Lincoln, Lynham, and Guba 2011). Codes were collated into categories and potential themes, and all data were collated as relevant to each theme (Braun and Clarke 2006). The potential for second interviews was included in the study design; however, the follow‐up interviews were not needed because (1) an overall sense of the concepts and themes was obtained from the initial interviews and (2) the primary author determined that little new information would be discovered in a subsequent interview (Rubin and Rubin 2011). Data saturation was determined through an iterative process using techniques such as memo writing, cross‐group comparative analysis (e.g., within single interviews, within pairs and between different pairs) and data triangulation from multiple sources, different data collection methods and two researchers in the analysis process (Boeije 2002; Braun and Clarke 2006).

#### 3.1.6 Ethical Considerations

On 26 July 2022, the University of Wisconsin–Madison Minimal Risk Research IRB determined the study met the criteria for exemption (ID: 2022–0771). The research information and consent document shared information about Dr. LeClair's reasons for conducting the research, and all participants were able to ask questions during the consent and enrolment process and at any time during their participation in the study.

## 4 Findings

### 4.1 Characteristics of Participants

Thirteen people enrolled in the study, including five public health nurses, five CBO representative partners and three public health nurses who enrolled without their community partners for partial data collection. Participant demographic information was not collected, although many volunteered their intersecting social identities during data collection, including Indigenous, Black, White, woman, man and members of the 2SLGBTQ+ community. Nurses enrolled in the study reported working to advance climate justice in academic or nonprofit settings.

In the narratives below, public health nurses are labelled ‘RN’ and CBO representatives are labelled ‘CBO’. Participant pairs are numbered 1–8, with the three solo nurses labelled ‘RN 5, 6, and 7’. Pairs 1, 2 and 8 addressed corporate climate pollution in fenceline communities. Pairs 3 and 8 promoted Indigenous sovereignty and multispecies justice in frontline and fenceline communities. Pairs 4, 5, 6 and 7 promoted climate resilience and mitigation in frontline communities.

### 4.2 Strategies

These results relate to the study's aim to describe nurses' and CBO representatives' collaborative strategies for advancing climate justice with frontline and fenceline communities. Table 1 summarises the collaborative strategies that were either implemented or planned by nurses and their community partners. Table 2 summarises the strategies nurses and their community partners suggested but did not implement. Except for *self‐care*, all the strategies described by participants deductively aligned with the interventions described within the public health intervention wheel (Schaffer and Strohschein 2019). Tables 1 and 2 provide the public health intervention wheel themes and definitions, the aligned strategies identified from the interviews, the level of influence from the social ecological model and the number of supporting excerpts from this study. Below are descriptions and examples of the implemented or planned collaborative strategies supported with five or more interview excerpts (see Table 1 for more details). Nurses and their community partners implemented strategies aligned with nearly all the themes identified in the public health intervention wheel, except for *screening, delegated functions and consultation* (Schaffer and Strohschein 2019).

**TABLE 1:** Summary of implemented or planned strategies.

Theme & definition^a^	Strategies	Social Ecological Model Level	N: Excerpts
**Disease and Health Event Investigation**: Systematically gathers and analyses data regarding threats to the health of populations, ascertains the source of the threat, identifies cases and others at risk and determines control measures.	Citizen science was a technique used by nurses and their community partners. This refers to public engagement in scientific research activities (Den Broeder et al. 2018).	Community	7
	Examples of citizen science strategies implemented by public health nurses and their community partners: ○ Air monitoring with youth as a part of a science camp. ○ Soil testing first with community members, then in collaboration with the State Department of Health.		
	Conducted a historical analysis of environmental covenant attached to the deed for a property improperly containing radioactive waste.	Policy	
**Case Finding**: Locates individuals and families with identified risk factors and connects them to resources.	Tested workers' boots for radioactive material using a Geiger counter.	Individual	1
**Outreach**: Locates populations of interest or populations at risk and provides information about the nature of the concern, what can be done about it and how to obtain services.	Flyering, canvassing and door knocking to inform community members about toxins in the air or water. Organised community‐based participatory research focus groups to understand community member's experiences and needs related to their overexposure to environmental hazards. Connecting community members with farmers' market food to address lack of access to healthy food options due to toxins in the soil.	Community	5
**Referral:** Makes a connection to necessary resources to prevent or resolve problems or concerns.	The public health nurse made professional connections with her CBO partner to help address local environmental hazards in the community.	Interpersonal	1
**Case Management:** A collaborative process of assessment, planning, facilitation, care coordination, evaluation and advocacy for options and services to meet client needs. It uses communication and available resources to promote safety, quality of care and cost‐effective outcomes.	Public health nurses and their CBO partners assessed community needs and provided resources. Examples included: Assessing the needs of and providing resources to an unsheltered community to increase access to healthy foods and other essentials. Assessing the dietary preferences and fresh food needs of a tribal community to improve a farmers' market and decrease exposure to food grown in toxic soil.	Community	7
**Health Teaching:** Sharing information and experiences through educational activities designed to improve health knowledge, attitudes, behaviours and skills.	Public health nurses and their CBO partners educated community members about the health impacts of climate change and environmental hazards. Examples included: Presenting at school science fairs about environmental hazards such as radioactive waste perpetuated by oil and gas industries. Organising information about ongoing community exposure to environmental hazards for the EPA administrator's visit. Nursing students teaching community families about gardening and herbalism. Presentations/trainings/webinars for community members about climate justice topics such as climate health impacts, water democracy and the public health impacts of environmental degradation on women, water and the land. Organising Native cooking classes to increase access to healthy food and foster spiritual connections with the food and the land. Organising health and wellness day with the local church to build trust and a sense of well‐being in the community.	Community	21
**Counselling:** Establishing an interpersonal relationship at an emotional level, with the goal of increased or enhanced capacity for self‐care and coping.	Organised Native language classes and storytelling as cultural therapy to foster spiritual interconnections within the community and the land.	Community	1
**Collaboration:** Enhances the capacity to promote and protect health for mutual benefit and a common purpose. Collaboration involves exchanging information, harmonised activities and shared resources.	Public health nurses and their CBO partners engaged in collaboration at the interpersonal, community and organisation levels.	Interpersonal	5
	Examples include: Interdisciplinary collaboration with scientists to study environmental toxins and community exposure. Implementing Earth‐grounded CBO/RN partnership practices. Going slow and building RN/CBO relationships at the speed of trust. Sharing the workload.		
	Demonstrating allyship and solidarity between frontline and fenceline communities. Sharing burden of the work among community members to advance climate justice. Creating community storytelling spaces. RN holds space for listening to community members when from outside the community. Disseminating research findings at community events. Fundraising for community events and other climate justice‐related projects.	Community	10
	RN is on CBO's board. Implementing community‐based participatory action research through universities. School of Nursing students' experiential clinical placed with the CBO. CBO worked with nursing organisation to define environmental harms as public health issues.	Organisation	14
**Coalition building:** Helps promote and develop alliances among organisations or constituencies for a common purpose. It builds links, solves problems and/or enhances local leadership to address health concerns.	The following examples demonstrate the coalition‐building strategies of public health nurses and their CBO partners: Engaging in partnership calls with coalition members. Connecting organisations to support each other and communities. Implementing practices that build trust in coalition meetings.	Organisation	5
**Community Organising:** The process by which people come together to identify common problems or goals, mobilise resources and develop and implement strategies for reaching the objectives they want to accomplish.	Community organising was a frequent strategy utilised by public health nurses and their CBO partners. Examples included: Organising with workers in the fracking industry. Organising a rally against a cracker plant. Public health nurse and community partner formed a CBO to protect community health from oil and gas. Organising climate justice events (e.g., summit). Organising clean water distribution Growing, preparing and/or distributing fresh food from gardens and farms.	Community	17
**Advocacy:** The act of promoting and protecting the health of individuals and communities by collaborating with relevant stakeholders, facilitating access to health and social services and actively engaging key decision‐makers to support and enact policies to improve community health outcomes.	Public health nurses and their CBO partners strategically advocated for policy and systems change. Examples included: Presenting citizen science project results to city officials. Writing editorials. Engaging with local, state and federal governments for drinking water protection. Fighting extractive industry. Researching occupational health policies.	Policy/Systems	11
	Engaging community stakeholders for fair water prices and philanthropic support.	Community	1
**Social Marketing:** A process that uses marketing principles and techniques to change target audience behaviours to benefit society as well as the individual.	Public health nurses and their CBO partners implemented several social marketing strategies, such as: Creating t‐shirts to combat disinformation from the government in community settings. Partnering with media to report on community events and give community members a platform to speak. Partnering with media to expose corporate climate pollution and state violence.	Community	3
**Policy Development and Enforcement:** Policy development places health issues on decision‐makers' agendas, establishes a plan of resolution, determines needed resources and results in laws, rules and regulations, ordinances and policies. Policy enforcement compels others to comply with the laws, rules, regulations, ordinances and policies created in conjunction with policy development.	The following are examples of policy strategies implemented by public health nurses and their CBO partners: Updating water sampling procedures to better monitor for environmental toxins. Drafting and getting signatures on letter for a smoke rule to protect farmworkers.	Policy	2
**Self‐Care:** engaging in practices that support personal resilience throughout the long struggle towards climate justice.	Public health nurses and their CBO partners discussed self‐care strategies, such as: Connecting with nature and other Earth‐grounded practices. Making time for rest. Receiving mentorship. Participating in support groups for community organisers. Taking intentional breaks from community organising. Making time for play. Prioritising relationships and collective care. Setting boundaries for self‐preservation.	Intrapersonal	15

^a^ Theme names and definitions, with the exception of *self‐care*, are taken from the public health intervention wheel (Schaffer and Strohschein 2019).

**TABLE 2:** Summary of suggested or not implemented strategies.

Theme & Definition	Strategies	Social Ecological Level	N Excerpts
**Disease and Health Event Investigation**: Systematically gathers and analyses data regarding threats to the health of populations, ascertains the source of the threat, identifies cases and others at risk and determines control measures.	Community Environmental Exposure Symptom Survey	Community	5
**Case Management:** A collaborative process of assessment, planning, facilitation, care coordination, evaluation and advocacy for options and services to meet client needs. It uses communication and available resources to promote safety, quality of care and cost‐effective outcomes.	Providing resources to meet the needs of focus group participants.	Community	1
**Health Teaching:** Sharing information and experiences through educational activities designed to improve health knowledge, attitudes, behaviours and skills.	Storytelling and outdoor signage to retain cultural memory of land trauma.	Community	1
**Collaboration:** Enhances the capacity to promote and protect health for mutual benefit and a common purpose. Collaboration involves exchanging information, harmonised activities and shared resources.	Schools of Nursing being a part of the climate justice movement.	Institution	2
	Create a climate justice nursing training centre to better partner with frontline/fenceline communities.		
**Coalition Building:** Helps promote and develop alliances among organisations or constituencies for a common purpose. It builds links, solves problems and/or enhances local leadership to address health concerns.	Create a transdisciplinary climate justice training centre for health professions.	Institution	1
**Community Organising:** The process by which people come together to identify common problems or goals, mobilise resources and develop and implement strategies for reaching the objectives they want to accomplish.	Integrating frontline community members into the organising efforts.	Community	1
**Advocacy:** The act of promoting and protecting the health of individuals and communities by collaborating with relevant stakeholders, facilitating access to health and social services and actively engaging key decision‐makers to support and enact policies to improve community health outcomes.	Local health department advocates for climate justice in solidarity with communities.	Policy/Systems	2
	Researcher presents climate injustice evidence to state officials for rule change.		
**Self‐Care:** Practices that support resilience through the long struggle towards climate justice.	Nurses protecting and advocating for themselves in their workplace.	Policy/Systems	2

#### 4.2.1 Disease and Health Event Investigation

Nurses and their community partners gathered and analysed data regarding threats to the health of frontline and fenceline communities. Corporate and industrial pollution worsened population health, including increased respiratory, bone, neurological and heat‐related diseases. To gather and assess data about these threats, nurses and their community partners implemented citizen science techniques, such as air and soil testing in the community.

The [STATE AGENCY], the [STATE] Department of Health came out and did soil sampling of the site, and they only did that because we citizens did it first and had it sent off to [COMPANY] in [NEARBY STATE], which is a nuclear testing facility. So anyway, we sent our soil samples to them, and then the [STATE] Department of Health came out and did their own soil sampling, and they found elevated levels in the public domain, like levels that were above a health‐based standard, and we asked the State and the U.S. EPA, ‘What are you going to do about it? Now it's proven that it's a problem. What are you gonna do?’ I just asked them that today, and it's been, like, it took a whole year for all this to happen.—RN.

#### 4.2.2 Outreach

Through *outreach* activities and *case finding*, nurses and their community partners identified frontline and fenceline community members at risk and provided them with information or services. Examples included canvassing and helping community members access clean water and healthy food.

… as soon as the water was shut off, you guys went out and bought as much water as you could for the group because it was either you guys bought it, or nobody was going to get it there. We had to bang on doors to let them know that the water was bad.—RN

We would also ask members who did come to the farmers market, ‘Do you know of people who need bags of food from the farm?’ They would offer to bring the bags of food themselves, whether it was a neighbor, whether it was a family member, etc., whether it was someone they could drive, you know, they could use their own car to drive to somewhere where someone didn't have the ability to drive to where the produce was at the farmers market, so it was just a work in progress, if you will, of getting the healthy food and getting the water and getting the PPE out to people who needed it, so that was very high priority on our list.—RN

#### 4.2.3 Case Management

Nurses and their community partners implemented *case management* strategies at the community level. This was done by assessing the needs of frontline and fenceline communities and providing necessary resources.

I was the one that was like, ‘We should probably ask what their needs are’. … ‘What else? What else do they need?’ … So, he did ask, and everybody was very, very grateful for the food. They loved it. It was all very delicious. They actually really liked having healthy food because the rest of the time, they did have, like, kind of the dregs of food in society, you know… And he was like, ‘Oh, there's actually a lot of need for other resources’. They were like, ‘We need socks and bandages, and we need water and batteries and gasoline’, and like all these things that you never even thought about.… Anyway, I'd like to say that I made it better [laughs] just by being like, ‘Hey, we should talk to the community.’—RN

#### 4.2.4 Health Teaching

Nurses implemented *health teaching* strategies with their community partners. These strategies involved sharing climate justice information and experiences through educational activities to improve health knowledge, attitudes, behaviours and skills.

I did a whole session on women, water, and wellness, and she (RN) was one of the speakers. And she talked about public awareness and public health. So, we did a public health campaign around water.—CBO

Health teaching was implemented at the community level, including presenting at community events, teaching government staff and politicians, having nursing students teach community members, holding health and wellness days and telling stories through filmmaking. Nurses and their community partners also educated youth, which gave them a sense of hope.

I think even something small like school science fairs and teaching kids, you know, what's in this stuff. Or that Radium‐226 is bad, you shouldn't be breathing it. I mean, the adults …until it's affecting them personally, economically, or physically, they're not going to care, but I think with everything, generationally, once kids start learning, and they carry that with them into their adult life that generations change things. I mean, we can look back from the forties on how, you know, each generation evolves from education and tolerance and, you know, each generation changes in some way. I think that, in this sense, is probably the only way because of capitalism and the amount of money that's invested into oil and gas that there's a possibility of change and making sure that the public's health is protected and that the environment is protected, is through generational education and change.—CBO

#### 4.2.5 Collaboration

Nurses and their community partners implemented interpersonal, community and organisational collaboration strategies. The partners implemented interpersonal strategies, including meeting outside to connect with the Earth and going slowly to build trust.

…it developed over time into a relationship, and it was a very different relationship. At one point, it was people in need, and it was academia, and slowly, over time, we've melted and gotten to this point. It's been a slow process, but you know, sometimes building relationships is a slow process, and it's been one that's been dependable, and it's been stable, and that's what our people need: something that is stable, right, and dependable. It doesn't have the immediate answers that we're looking for, but we see that it could have something in the future that would be beneficial to the survival of our people.—CBO

Community collaboration strategies included demonstrating allyship and solidarity, sharing the work's burden, creating community storytelling spaces, holding space for listening to community members, disseminating research findings at community events and fundraising activities.

I have been very surprised, in a good way, at how many people there are who are very passionate about the work we do with advocacy, supporting families, you know, anti‐violence work, planetary health work. I met [CBO] that way through [NAME], who runs [ORGANIZATION]. It's really been through this journey of probably the last four, three to four years, mostly that I've really allowed myself to open up more and collaborate more.—RN

Organisational collaboration strategies included the nurse and CBO partners joining each other's boards, implementing community‐based participatory action research partnerships, holding Schools of Nursing students' experiential clinicals with the CBO and CBOs collaborating with nursing organisations to define environmental harms as public health issues.

The environmental nurses that I work with from DC and throughout this whole [State] have committed themselves to putting a face of public health on environmental harms and disparities.—CBO

Thank you for doing this important work and bringing awareness to how important academic‐community partnerships are for me in particular, but also nurses and other agencies because our work goes further when we collaborate together.—RN

#### 4.2.6 Coalition Building

Nurses and their CBO partners helped promote and develop alliances among organisations or constituencies for climate justice. Strategies included engaging in partnership calls with coalition members, connecting organisations to support each other and communities and implementing practices that build trust in multiorganisation meetings.

[RN] joins our monthly partnership calls. The partnership calls serve as just a moment to connect, collaborate on the work, link to projects that community partner organizations are doing, as well as, you know, potentially we can collaborate on projects.—CBO

And the collaboration ‐ there's so many, there's such a good network, I mean, this is one organization that kind of was the lead with what I was doing. But I came across so many other organizations that were networked in with this organization and that work together, and you know, how strong their communication is, and it just comes naturally when they're part of the community, and they see the need in front of them every day.—RN

#### 4.2.7 Community Organising

*Community organising* is the process by which people come together to identify problems or goals, mobilise resources and develop and implement strategies for reaching the objectives they want to accomplish (Schaffer and Strohschein 2019). Examples of ways nurses and their community partners engaged in community organising include forming and organising the CBO and organising workers, rallies, events and access to clean water and fresh food.

I just started to educate myself, and you know, just learn about it. And from there I led a community group here. It really started with me and my dad because he's like on the same wavelength as me, as far as trying to use our voice to wake people up. And you know there's not too many people that are activists here in the [REGION]. So, we started it, and we started going to council meetings about this facility. And it wasn't just like we believe this is a problem. It really is a problem, and it's all … I know that you guys are focused on other things, not just climate, but it is climate‐related ‐ it all is … but it's also public health, it's also environmental justice and the future ‐ the future of this [REGION]. So, anyway, started out with me and him and then from there, we started to garner more support from the community. We had a small group of about 10 or 12 people that were meeting regularly, and it was really starting to pick up steam, and we got a lot of media attention.—RN

#### 4.2.8 Advocacy

Nurses and their community partners advocated for promoting and protecting the health of frontline and fenceline communities by collaborating with relevant stakeholders, facilitating access to health and social services and actively engaging key decision‐makers to support and enact policies to improve community health outcomes.

What agency are we not hassling? It's everybody. It's [AGENCY] which is the [STATE] Department of Natural Resources. They regulate oil and gas ‐ they don't really regulate. The [STATE] Environmental Protection Agency, which is an arm of the U.S. Environmental Protection Agency… now that we have a President who cares more about the environment, he put people in charge that are listening to environmental justice concerns, whereas the previous administration did not whatsoever, I don't think. This one we're working with the U.S. EPA, which the [REGION]Administrator came down to talk to us in person, like the head lady herself, and that was huge. I mean that doesn't happen. That just doesn't happen, especially around here. So that was a big thing, and that's because, you know, Biden. So those three, and then there's, I mean, the [STATE] Department of Health, which they're out to lunch, too. They're not really… we're not really working to advocate to them. They just play a role in this situation, which I can talk about. So, there's four agencies, and the balls just bouncing around like chaotically, really, but yeah, there's a lot.—RN

Nurses and their community partners advocated for climate justice by presenting citizen science results from air and soil testing to city officials, writing editorials, advocating for drinking water protection, speaking out against extractive industries such as fracking and advocating for agricultural worker health by researching occupational health policies.

Then we did work on COVID, and you know, she was part of why we got in touch with our governor, [STATE GOVERNOR]. He was the first state to say, we're going to do a moratorium on water shutoffs during this COVID pandemic. Because what are you doing—what are you doing shutting water off? And you're saying the very thing that can save people's lives is to wash their hands, but people are living without their water. So [RN] became part of the [STATE] Energy Table that not only lifted water, but also lifted the disproportionate connection to energy, solar‐renewable energy and climate justice.—CBO

#### 4.2.9 Self‐Care

The thematic analysis of the transcripts utilised the public health intervention wheel in deductive analysis. *Self‐care* was the only theme that arose inductively from the data and was not found in the public health intervention wheel. Participants described *self‐care* as engaging in practices that support personal resilience while they navigated the transition from climate injustice to climate justice. Examples of the *self‐care* strategies that nurses and their community partners implemented included connecting with nature, making time for rest and play, receiving mentorship, participating in support groups, taking breaks from community organising, prioritising relationships and collective care and setting boundaries for self‐preservation.

Well, one of the things I have always said to nurses, and, you know, when I was still working at my prior job as a school nurse, in speaking to staff, it's always about self‐care. If you are not doing good self‐care, you cannot care for others. So, one of the critical things for me is eating well, exercising, doing things to de‐stress, such as yoga, exercise is a de‐stressor for me, some people find exercise stressful, I don't, but, also, having good quality time with my family, and my dog. I do really good self‐care for myself with family, with my dog, with exercising, and with staying positive. I think staying positive is so incredibly critical, and I always try to look at the glass half‐full. I never want to see the glass half‐empty, because that doesn't benefit me in any way, shape, or form, and so, I feel that, where there's hope, there's always the propensity to move forward in a positive direction and make change. I think, if I were to think about the overwhelming nature of the plight of the [NATIVE NATION], I think it would almost paralyze me, and I refuse to let that happen, and they certainly haven't let it paralyze them. Their resilience is absolutely incredible, and I think one of the things that I‐ that helps me move forward each day with working with them is seeing their resilience, and they propel me forward because of who they are and because of their positiveness.—RN

## 5 Discussion

This study is the first to describe the collaborative strategies nurses and their CBO partners implemented to support the transition from climate injustice to justice in frontline and fenceline communities in the United States. Findings from this study indicated that nurses and their CBO partners work to transition communities away from systems of extraction towards local and regenerative systems that support resilience. These findings align with recommendations from climate justice groups (International Climate Justice Network 2002; Miller and Sisco 2002; ‘Mobilization for Climate Justice: Open Letter to the Grassroots’, 2009; Principles of Environmental Justice 1991; The Just Transition Alliance 1997).

Many strategies that participants in this study implemented also aligned with the principles listed in the Global Nurse Agenda for Climate Justice, including but not limited to *Principle #3*—expose corporate actions that maintain climate injustice; *Principle #10*—monitor ecological assessment data on human health risks; *Principle #13*—affirm the sacredness of nature, ecological unity and interdependence of all species; *Principle #23*—organise for infrastructure system change (food, water, air and earth) through participatory action approaches; and *Principle #29*—participate with communities in climate justice actions through assessment, planning, activism, mitigation, adaptation and restoration (Evans‐Agnew, LeClair, and Sheppard 2024). This study provides further evidence in support of these principles and suggested strategies in the Global Nurse Agenda for Climate Justice.

All participants implemented strategies aligned with the public health intervention wheel, providing further evidence for public health nurses to utilise in practice (Schaffer and Strohschein 2019). The results from this study also provide evidence for competencies listed under Standard 17 in the ANA Public Health Nursing Scope and Standards of Practice (ANA 2022). These competencies focus on the public health nurses' role in promoting and protecting environmental health, planetary health and environmental justice. Finally, the findings from this study support the ANA's recent position statement on the *Nurses' Role in Addressing Global Climate Change, Climate Justice, and Planetary Health* (2023). The statement guides nursing interventions in any setting through research, education, advocacy and practice. A common recommendation in the previously described documents, supported by this study's findings, is the critical need for nurses to collaborate with CBOs when advocating for justice in frontline and fenceline communities (ANA 2022, 2023; Schaffer and Strohschein 2019).

This study's findings align with the science and field of planetary health (Faerron Guzmán et al. 2021). While participants were asked about climate justice, some explicitly identified their work with planetary health or discussed the intersecting crises of climate change, pollution and multispecies injustice (e.g., biodiversity loss). These perspectives align with the United Nations Environment Programme's focus on the ‘Triple Planetary Crisis’, which includes the intersecting health impacts of climate change, pollution and biodiversity loss (United Nations Environment Programme 2021).

### 5.1 Limitations and Strengths

This study included a small sample of nurses from academic and nonprofit settings in a few regions across the United States. Therefore, the results do not necessarily apply to nurses working in other settings or countries, and additional or different thematic findings might emerge from participants in more diverse geographic settings. However, climate justice is internationally relevant, and this research can inform nurses' ability to pursue climate justice locally and globally. Although many participants freely disclosed their positionalities, such as their race and gender, they were not directly asked to share their demographic information, thereby potentially limiting the data analysis and interpretation of study findings.

### 5.2 Recommendations for Further Research

This study holds many implications for future research. Very few previous studies have explored specific, evidence‐based strategies for health professionals to address the effects of climate change, and none address climate injustices in frontline and fenceline communities (Dupraz and Burnand 2021; LeClair, Watts, and Zahner 2021; Maibach, Frumkin, and Ahdoot 2021; Schenk et al. 2019). Nurses and their community partners in this study provided essential evidence of the types of climate justice strategies they implemented across individual, interpersonal, community, policy and systems levels. However, as previously described, the participant sample in this study was small and represented nurses in academic and nonprofit work settings across seven US states. Nurses' engagement in the climate justice movement through diverse employment and geographic settings must be better understood to develop effective strategies that improve population health. These findings highlight the need to consistently measure nurses' and CBO representatives' work across more diverse employment and geographic settings to identify critical gaps for intervention. Creating a nationwide survey instrument would be helpful to standardise the descriptions of strategies so they could be measured and compared across settings. This work could inform the development of evidence‐based nursing practice and informed decision‐making in planetary health. Finally, measuring short‐, medium‐ and long‐term outcomes of implementing the collaborative strategies was beyond the scope of this study. Future research to evaluate whether and how collaborative strategies produce outcomes is needed to build evidence to guide the choice and implementation of future nurse/CBO representative collaborative strategies in communities.

### 5.3 Implications for Policy and Practice

Findings from this study support nursing engagement in policy work to advance planetary health. Engagement in advocacy for policy enforcement provided opportunities for study participants to hold governments and corporations accountable for perpetuating health inequities in frontline and fenceline communities. Participants engaged with multiple government agencies to promote health equity and climate justice. Through their collaborative partnerships, they recognised planetary heath threats and proposed practical solutions.

**FIGURE 1:**
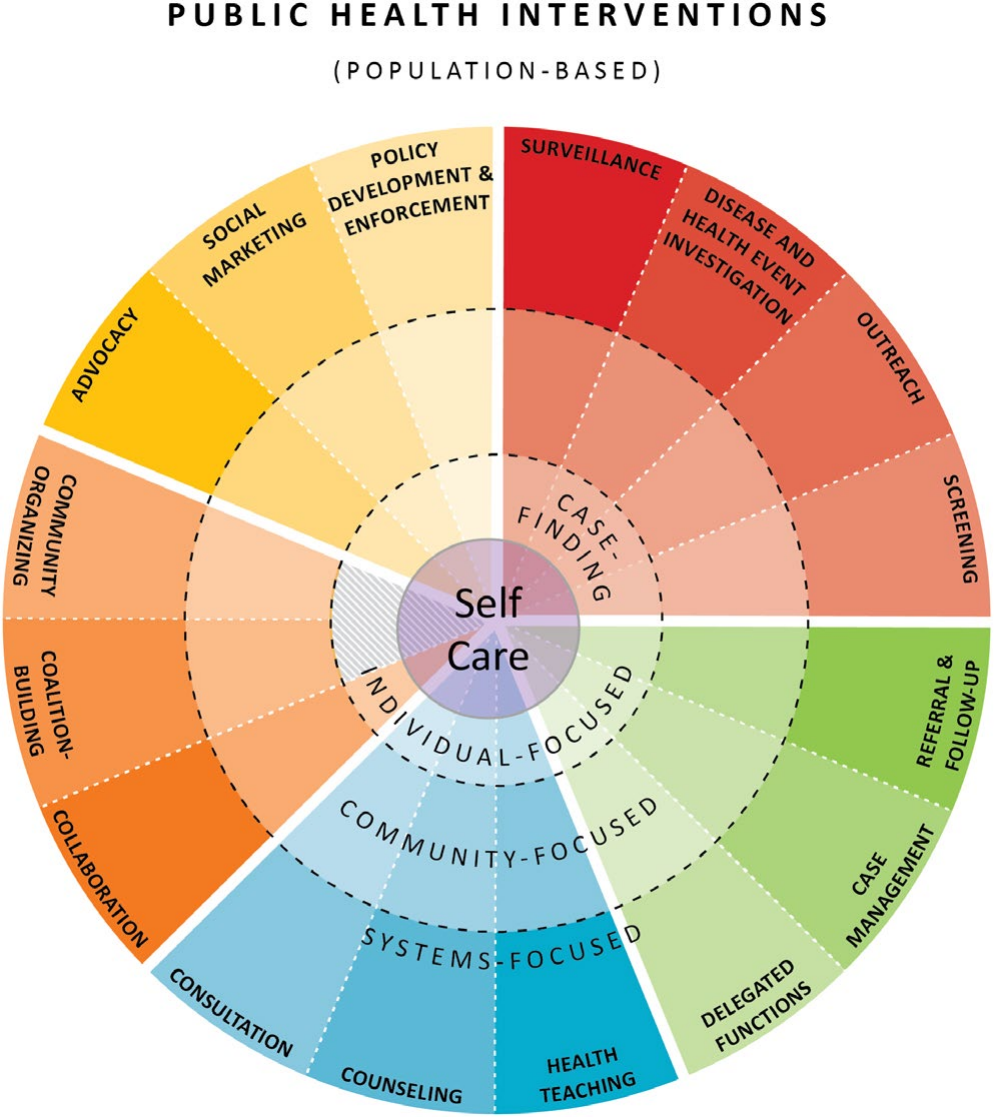
Adapted public health intervention wheel for climate justice.

This study supports using the public health intervention wheel to help guide nurses in their planetary health practice (Schaffer and Strohschein 2019). The public health intervention wheel could be adapted to emphasise *self‐care* throughout the wheel as the hub—the central support (Figure 1). Nursing practice could incorporate strategies listed in the *self‐care* ‘hub’ of the wheel to help sustain individual and collective resilience throughout the long struggle for justice. This study also supports nurses to take collective action with their community partners to address the effects of colonisation, such as promoting food sovereignty within Indigenous frontline and fenceline communities. Addressing the effects of colonisation is a new competency for public health practitioners in the United States (Council on Linkages Between Academia and Public Health Practice 2021).

## 6 Conclusion

Nurse and CBO representative collaborative strategies included investigating disease and health events; identifying individuals, families and populations at risk and connecting them with resources; providing health teaching and counselling; organising communities and coalitions; and advocating for policy development and enforcement. Self‐care was integral to nurse–community collaborative strategies to support resilience and well‐being in the long struggle for climate justice. Nurses and their community partners recognised the importance of an expansive understanding of climate justice to include intersections of pollution and multispecies justice in promoting public and planetary health equity.

## Author Contributions

All authors have agreed on the final version and meet at least one of the following criteria (recommended by the ICMJE*):Substantial contributions to conception and design, acquisition of data or analysis and interpretation of data; Drafting the article or revising it critically for important intellectual content.

## Conflicts of Interest

The authors declare no conflicts of interest.

## Supporting information


Data S1.

## Data Availability

The authors have nothing to report.
