# Supplementary material for: Climate Justice Strategies Implemented by Public Health Nurses and Their Community Partners
Source: J Adv Nurs. 2024 Nov 11;81(12):8316–30. doi: 10.1111/jan.16598 (PMC12623680; doi:10.1111/jan.16598)
Supplement: Supplementary file 1 — Data S1. [file JAN-81-8316-s001.pdf]

Interview Guides

Interview Guide 1: Participatory Photomapping (PPM)

This PPM interview with each PHN/CBO pair will address *Aim 1: Describe how PHNs and their CBO partners envision and experience climate justice*. We will use PPM to understand what climate justice means to participants. Each PPM interview will take 1-2 hours.

The method that will be used is the **SHOWeD** technique (Wang & Pies, 2004), where the facilitator asks the participants the following questions to get more information about the photographs:

S: What do you **see** here?

H: What really **happened** or is **happening** in the picture?

O: How does this relate to **our** (e.g., community/society) lives**?**

W: Why does this situation (problem, concern, or strength) exist?

Who is responsible for silencing or promoting our (e.g., community/society) voice? (Evans-Agnew et al., 2017)

D: What can we **do** about it?

Additional question: What do you **not** see in your picture (what is missing)? This question is guided by the Critical Environmental Justice Nursing for Planetary Health Framework (LeClair et al., 2021), which leads the participants to reflect on bias and what is missing in the picture.

The PHN and CBO will be encouraged to ask each other additional questions throughout the interview.

Interview Guide 2: Semi-structured Individual Interviews

This semi-structured interview will address *Aim 2: Describe strategies for addressing climate justice*. We will use semi-structured interviews to describe how PHNs and their CBO partners design, plan, and utilize their climate justice strategies, manage barriers/conditions, and work with facilitators. The interview will also address *Aim 3: Describe partnership processes to address climate justice.* We will use semi-structured interviews to understand the processes for effective PHN and CBO partnerships.

We will interview each PHN and CBO separately. Initially, open-ended, generative questions will be used, such as, "Tell me about how you came to practice in the community." The guide will consist of two levels of questions: 1) main themes and 2) follow-up questions (Kallio et al., 2016). The main themes will cover the aims of the study, and participants will be encouraged to speak freely about their perceptions and experiences. Possible second-round interview questions will become more structured in response to emerging themes and linkages among concepts that need to be verified with new data because of the provisional nature of the themes. The same questions listed below will be asked of each PHN and CBO. Interviews will take approximately 1 hour.

First Round Interview Questions for PHNs and CBOs:

Framing statement: *Thank you so much for your time today. This interview will have three parts. The first part will be about the work you do and how you chose it, then we'll talk about your partnership with _______ [PHN/CBO partner], and finally, we'll discuss the challenges of working within systems that contribute to injustices.*

Your work:

1. Tell me about how you chose to do work that is based in the community.

The partnership:

2. How did you get involved in the partnership with ___________ [PHN/CBO partner]?

3. Tell me a bit about how you worked together.
    a. How did you communicate?
        i. Did one partner take the lead?
    b. What worked well?
    c. What were challenges?
        i. How did you work through the challenges?
    d. What decisions did you have to make together?
        i. How did you make them?
        ii. When did you make them?
    e. Did you have an opportunity to evaluate the partnership?
        i. If so, how?
        ii. If not, how might you?

4. Did anything surprise you in your work together?

5. What were the sweet spots in your partnership?
    a. What contributed to your positive relationship?
    b. What personal or professional resources supported your partnership?

6. Can you think of anything that was missing in the partnership that you'd like to see?

a. If yes, explain what was missing.
b. Were there any barriers?

Framing Statement: *Sometimes people who work in communities on issues of climate justice find that the rules and structures of the systems they are working in contribute to injustices and inequities. This can be frustrating.*

Working within unjust systems:

7. Did you find that systems were contributing to injustices?
    a. What does it feel like when you encounter a system of injustice?
    b. How do you keep going?
    c. Why do you keep going?

8. Is there anything that I did not ask you about that you would like to share?

Second Round Interviews: as needed for individual follow-up questions. Second-round interviews will take approximately 30 min-1 hour.

References

Evans-Agnew, R., Boutain, D., & Rosemberg, M.-A. (2017). Advancing nursing research in the visual era: Reenvisioning the photovoice process across phenomenological, grounded theory, and critical theory methodologies. *Advances in Nursing Science*, *40*(1), e1-15. https://doi.org/10.1097/ANS.0000000000000159

Kallio, H., Pietilä, A. M., Johnson, M., & Kangasniemi, M. (2016). Systematic methodological review: developing a framework for a qualitative semi-structured interview guide. *Journal of advanced nursing*, *72*(12), 2954-2965.

LeClair, J., Luebke, J., & Oakley, L. D. (2021). Critical Environmental Justice Nursing for Planetary Health: A Guiding Framework. *Advances in Nursing Science*. ePrint 1-10, doi: 10.1097/ANS.0000000000000398.

Wang, C. C., & Pies, C. A. (2004). Family, maternal, and child health through photovoice. *Maternal and child health journal*, *8*(2), 95-102.
